# Correlation Between Molecular Genetic Analysis and Nuclear Pleomorphism in Long-Term Recurrent and Metastatic Chordoma

**DOI:** 10.3390/cancers18060898

**Published:** 2026-03-11

**Authors:** Sarah Rebecca Ullmann, Julian Schreier, Juan Carlos Alberto Uribe Caputi, Marilena Georgiades, Joana Maria Ullmann, Christoph H. Lohmann, Martin Röpke, Denny Schanze, Sabine Franke, Franziska Sabrina Karras, Albert Roessner

**Affiliations:** 1 Institute of Pathology, Otto-von-Guericke University, 39120 Magdeburg, Germany; sarah.ullmann@ovgu.de (S.R.U.);; 2 Molecular Immunology and Epidemiology, Universidad Autónoma de Bucaramanga UNAB, Bucaramanga 680003, Colombia; 3 University Clinic for Radiology and Nuclear Medicine, Otto-von-Guericke University, 39120 Magdeburg, Germany; 4 Institute of Pathology, University Medical Centre Rostock, 18057 Rostock, Germany; 5 University Orthopedic Clinic, Otto-von-Guericke University, 39120 Magdeburg, Germany; 6 Institute of Human Genetics, Otto-von-Guericke University, 39120 Magdeburg, Germany

**Keywords:** chordoma, recurrences, metastases, nuclear morphometry, TMB, WES, immunohistochemistry

## Abstract

**Background/Objectives**: Recurrences and metastases occur frequently in chordoma and are the main factors influencing overall survival. However, prognostic biomarkers for recurrence remain limited. This study examines whether quantitative nuclear morphometry can capture recurrence evolution and whether it aligns with immunophenotype and genomic profiling. **Methods**: A total of 26 specimens from 12 adults (8 patients with non-recurrent tumors and 4 patients with multiple long-term recurrences and metastases over 7 to 16 years) were analyzed using whole-exome sequencing, immunohistochemistry, and nuclear morphometry. **Results**: Imaging studies and routine histology showed no consistent differences between groups. Morphometry revealed substantial intertumoral variability among non-recurrent tumors and significant longitudinal nuclear remodeling throughout recurrences, dominated by increased nuclear size and asymmetry, as well as altered shape. Primary tumors from patients who later recurred had smaller, more asymmetric, and denser nuclei than non-recurrent tumors. Recurrent samples showed higher proliferation and decreased lamin A/C expression, with focal disruption and detachment from the nuclear envelope in pleomorphic nuclei. The tumor mutational burden was low overall, varied between patients and timepoints, and tended to be higher in recurrent cases. **Conclusions**: Quantitative nuclear morphometry, integrated with immunophenotyping and genomic profiling, captures recurrence-associated phenotypic remodeling in chordoma and may provide a quantitative framework for future digital pathology or AI approaches, pending validation in larger cohorts.

## Simple Summary

Chordomas are rare malignant tumors with high recurrence rates and scarce treatment options due to chemo- and radiation insensitivity. At present, it is difficult to predict which tumors are likely to recur because routine light microscopy and imaging do not reliably capture subtle biological changes. We measured tumor cell nuclei in primary tumors, long-term recurrences, and metastases using quantitative image-based methods. We compared the results with protein expression and genome-wide mutation load obtained from whole-exome sequencing. Recurrent tumors showed measurable pleomorphic changes and higher proliferation over time. Loss and disruption of nuclear envelope proteins were observed in highly irregular nuclei of recurrent and metastatic tumors. These results suggest that objective nuclear measurements, combined with molecular profiling, may help identify aggressive disease courses and provide a foundation for the development of automated, AI-assisted digital tools for future risk assessment.

## 1. Introduction

Chordomas are rare malignant bone tumors mostly affecting the axial skeleton and skull base [1]. The tumorigenesis of chordoma remains incompletely understood, though it is presumed to arise from undifferentiated remnants of notochordal cells in adults or from a benign notochordal cell tumor (BNCT) [2]. Histologically, chordomas are classified as conventional, dedifferentiated, or poorly differentiated [3]. Conventional chordomas are typically composed of physaliphorous cells within a myxoid stroma and are arranged in lobules separated by fibrous septa [4]. Some tumors exhibit necrosis, hemorrhage, or BNCT-like components [5]. Although chordomas are slow-growing and could biologically be considered low-grade tumors [6], they can be compared to sarcomas in terms of aggressiveness due to their high multifocal recurrence rate of up to 65% and risk of metastasis [7,8,9].

Pleomorphic changes in the form and structure of nuclei and nucleoli, as well as in tissue architecture, can be characteristic of tumor progression or recurrence in solid tumors [10]; such changes have been recognized as key criteria for tumor grading [11], though qualitative assessments often show high interobserver variability [12]. Thus, quantitative analysis of histopathological findings has been established as a complementary diagnostic tool in numerous malignancies [12,13], including breast cancer [14], urothelial neoplasms [15,16,17], malignant melanoma [18], colorectal cancer [19], myxoid liposarcoma [20], and mantle cell lymphoma [21]. Morphometry has been successfully used to distinguish between benign and malignant thyroid lesions [22]. Several studies have evaluated nuclear dysregulation in recurrences or metastases across other solid tumors [23]. Changes in histological grade based on nuclear morphometry have been observed in locally recurrent soft tissue sarcomas [24]. Furthermore, nuclear morphometry has been applied to predict recurrence in osteosarcoma [25] and ductal carcinoma in situ of the breast [26], as well as to differentiate between primary and metastatic lesions in Wilms’ tumors [27]. However, no morphometric studies dedicated to recurrent chordoma have been published to date, although nuclear pleomorphism has been discussed as a prognostic factor in conventional chordoma [28,29].

Histological abnormalities are the main criteria for histopathological diagnosis of malignancy [30], with size, shape, chromatin structure, and irregularities of the outer nuclear membrane considered [31,32]. However, investigations rarely integrate morphological abnormalities with molecular genetic alterations [22,33]. Sarcomas generally exhibit a lower tumor mutational burden (TMB) than other carcinomas [34]. Furthermore, carcinomas can show considerable genomic evolution in recurrences and metastases [35]. In contrast, little is known about the long-term genomic evolution of other mesodermal tumors such as chordomas [36,37]. Specifically, it remains unclear whether the tumor mutational burden (TMB) and other measures of mutational accumulation are associated with reproducible changes in nuclear morphometry and immunophenotype in recurrent chordoma [38].

This study investigates the association between histological and immunohistochemical findings, quantitative nuclear pleomorphism, and molecular measures of tumor mutational load, particularly the TMB, in long-term recurrent versus non-recurrent chordomas with the following questions:

1. Do non-recurrent tumors exhibit significant differences in morphometric parameters?
2. Do tumors with multiple long-term recurrences and/or metastases show measurable changes in pleomorphism over time? Do levels of lamin A/C, which are key filament proteins of the nuclear envelope, change correspondingly?
3. Do primary tumors from patients who later developed recurrences differ from non-recurrent tumors at baseline?
4. Are nuclear morphometric changes associated with molecular measures of mutational load, particularly the TMB?

The objective is to characterize morphometric, immunophenotypic, and molecular evolution over time through the relationship between morphonuclear and genetic alterations, thereby contributing to a more comprehensive understanding of recurrence development in chordoma. These findings are expected to provide some insights into therapeutic options and inform future studies on therapeutic vulnerabilities in long-term recurrent chordoma.

## 2. Materials and Methods

### 2.1. Sample Selection

**Figure 1:**
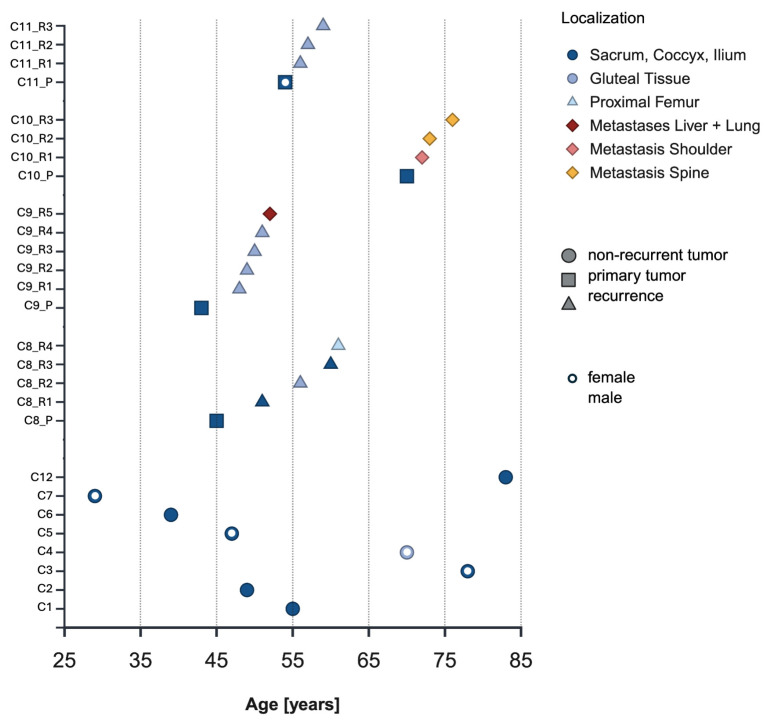
Clinical information for all patients is visualized over time, with each data point colored to indicate the tumor’s localization. Cn, case number; Rn, recurrence number. Patients with non-recurrent tumors have a heterogeneous age distribution, with a mean of 52 ± 18.61 years. Patients who later developed multiple long-term recurrences were 53 ± 12.30 years old at the time of diagnosis and had 3.5 recurrences on average over a time span of 7 to 16 years.

This is a descriptive case series study of tissue samples obtained from patients with non-recurrent chordomas and those with multiple long-term recurrences and/or metastases, in whom histological features, immunohistochemical profiles, nuclear pleomorphism, and the TMB were evaluated. A total of 26 formalin-fixed, paraffin-embedded (FFPE) tissue samples from 12 patients were retrieved from the histopathological archives of the Bone Tumor Registry of the Institute of Pathology of the Otto-von-Guericke University, Magdeburg, Germany. Of the 12 patients, 8 had primary tumors without recurrences, whereas 4 developed multiple long-term recurrences and/or metastases. Seven out of eight patients with non-recurrent tumors were deceased at study initiation, and the remaining patient had a follow-up of six years. Clinical data were provided by the Departments of Orthopedic Surgery and Radiotherapy. The clinical data, summarized in Figure 1, included age, sex, tumor site, treatment regimens (radiotherapy/chemotherapy), and, when available, the timing of long-term recurrences. This study was approved by the Ethics Committee of the Medical Faculty of the Otto-von-Guericke-University of Magdeburg, Germany (Approval No. 132/21; 27 August 2021).

### 2.2. Histopathological Analysis

An experienced bone pathologist (A.R.) reviewed all slides, evaluated the histology, and classified the tumors according to the current WHO classification of Tumors of Soft Tissue and Bone (5th edition). Only conventional sacral chordomas in adult patients were included in this study.

### 2.3. Immunohistochemistry

Immunohistochemical staining was performed on all samples for brachyury (EPR18113, Abcam limited, Cambridge, UK), Ki-67 (MIB-1, Dako Corporation, Agilent, Santa Clara, CA, USA), p53 (453M-95, DO7, Cell Marque, Rocklin, CA, USA), S100 (Z0331, Dako Corporation, Agilent), vimentin (Vim384, Dako Corporation, Agilent), E-cadherin (EP700Y, Cell Marque, Darmstadt, Germany), pancytokeratin (PCK, AE1/3, Leica Biosystems, Nußloch, Germany), EGFR (E30, Dako Corporation, Agilent), VEGF (VG-1, Santa Cruz Biotechnology, Dallas, TX, USA), SMARCB1 (25/BAF47, BD Biosciences, Franklin Lakes, NJ, USA), lamin A/C (EP4520-16, Abcam Cambridge Biomedical Campus, Cambridge, UK), PD-1 (315M-95, Cell Marque, Rocklin, CA, USA) and PD-L1 (M3653, Dako Corporation, Agilent). Formalin-fixed, paraffin-embedded (FFPE) tissue sections (2 μm) were dewaxed in xylene and rehydrated through graded ethanol, followed by antigen retrieval using EDTA buffer (1 mM; pH and retrieval conditions according to the laboratory standard protocol). Staining was performed using the automated immunohistochemistry slide-staining system, Ventana NexES (Ventana Medical Systems, Darmstadt, Germany). Sections were incubated with the respective primary antibodies at 37 °C for 30 min. Staining intensity was assessed semi-quantitatively and recorded as negative, weak, moderate, or strong based on visual evaluation by a pathologist. The Ki-67 index was determined via manual counting in 2–6 randomly selected high-power fields per slide (400×) and is expressed as the percentage of positively stained tumor nuclei. An average of 1764 nuclei were analyzed per sample (44,107 nuclei in total).

The p53 index was determined analogously via manual counting in one randomly selected field per slide (200×) and is expressed as the percentage of tumor nuclei with strong staining. In total, 24,478 nuclei were counted (mean of 1064 per sample).

### 2.4. Morphometry

Nuclear morphometry was assessed by measuring a mean of 317 ± 15 nuclei per sample on H&E-stained slides at 600× magnification using NIS Elements D Software (Nikon Instruments Inc., Melville, NY, USA; v4.20.03). Nuclei were sampled using a systematic field-selection approach. After identifying representative tumor areas at low magnification, fields of view were selected by moving the mechanical microscope stage in a predefined stepwise pattern (cross-table) across the tumor region, avoiding necrotic, hemorrhagic, or artifact-prone areas. Within each selected field, tumor cell nuclei were traced manually on digitally captured images using a mouse, and the results were tabulated in Excel. A minimum of 300 nuclei per sample were traced; within each selected field of view, all eligible tumor nuclei were traced consecutively to minimize subjective selection. As an internal quality-control step, duplicate sections from the same specimen were analyzed alongside all other samples and yielded highly comparable morphometric summary statistics (Bland–Altman bias of 0.14 µm^2^). To avoid pseudo-replication, only one replicate per specimen was randomly selected for inclusion in the main analysis. In addition to the nuclear area, further morphometric parameters were assessed to characterize differences in nuclear features between non-recurrent and recurrent/metastatic cases. These included size, shape, texture, and density descriptors. The methodological framework was adapted from Khatri et al., who used morphometric analysis to distinguish between benign and malignant thyroid lesions [22].

### 2.5. Whole-Exome Sequencing

Whole-exome sequencing (WES) is widely used to determine TMB and provides broader genomic coverage than targeted panels [39,40]. The TMB is commonly defined as the number of nonsynonymous somatic variants per megabase of callable coding sequence [41]. Thus, the mutational profiles in this cohort were analyzed through WES.

Prior to DNA extraction, tumor areas were marked on 2 µm H&E-stained FFPE slides by an experienced pathologist and traced on 6 µm slides under 100× magnification using a VWR VisiScope microscope (VWR International bvba, Leuven, Belgium). Marked tissue was macrodissected prior to deparaffination following the standardized protocol of the Laboratory for Molecular Diagnostics of the Institute of Pathology in Magdeburg, Germany. Subsequently, DNA was isolated using a NucleoSpin^®^ Tissue DNA purification kit from Machery-Nagel GmbH, Düren, Germany, following the manufacturer’s instructions. The extracted DNA was quantified using a Qubit Fluorometer (Invitrogen Thermo Scientific by Life Technologies, Eugene, OR, USA) and a Qubit™ dsDNA HS Assay Kit and stored at −20 °C. The SureSelect XT HS2 DNA System was used for DNA library preparation and target enrichment, and the Illumina platform from Agilent Technologies (Santa Clara, CA, USA) was used for enzymatic fragmentation, library preparation, and hybridization capture, as well as post-capture sample processing for multiplex sequencing, following the manufacturer’s protocol (vD0, April 2021). DNA quality and quantity were assessed regularly between workflow steps. The captured libraries were stored at −20 °C before sequencing on an Illumina NextSeq^®^550 at the Institute for Human Genetics of the University Hospital Magdeburg, Germany (Medical Faculty, Otto-von-Guericke University).

The sequenced data were processed using the Varvis^®^ pipeline (Varvis variant analysis v1.21.2, limbus-medtec, Rostock, Germany) [42]. They were aligned to the GRCh37 reference genome for variant calling using the following filters to reduce low-level subclonal calls: minor allele frequency (MAF) Gnom total of under 0.001, variant allele frequency (VAF) of ≥0.1, reads-index ≥ 40, impact “high” or “moderate” (as annotated by Varvis), and AltAF index ≥ 0.15. Amino acid changes that did not alter the protein product (synonymous variants) were excluded. Putative germline polymorphisms were filtered using dbSNP and the internal Varvis database. Variants observed in ≥100 entries in the internal database were excluded (AllexesFound ≥ 100). The TMB was calculated as the number of nonsynonymous SNVs + InDels per 35 Mb of callable exonic sequence.

### 2.6. Statistical Analysis

All statistical analyses were performed using MATLAB (The MathWorks Inc. v9.9/R2020b) and Microsoft Excel (Microsoft Corporation, v16.95.4 (25040241)). The Shapiro–Wilk test was used to assess normality, supported by a visual inspection of individual histograms and Q-Q plots. Variance homogeneity was assessed using Levene’s test and visualized with boxplots. Because multiple nuclei were measured per specimen, the specimen was defined as the primary statistical unit. Nuclear-level measurements were summarized as specimen-level medians, whereas nucleus-level distributions were used for visualization only. Intertumoral variability among non-recurrent tumors was assessed descriptively using the range, interquartile range (IQR), and the between-tumor coefficient of variation $\left(\left(\frac{standard\;deviation}{average}\right)\times 100\right)$ calculated across specimen-level medians. Given that only one specimen per non-recurrent case was available, no inferential hypothesis testing between individual non-recurrent cases was performed. Changes within recurrent cases were treated as dependent measurements and explored using non-parametric repeated-measures approaches (Friedman test) with Bonferroni-adjusted post hoc pairwise comparisons. Given the small number of independent patients and unequal numbers of timepoints, these longitudinal *p*-values were interpreted as exploratory. The Mann–Whitney U test was employed to compare non-recurrent tumors (NRTs) with primary tumors from recurrent cases based on specimen-level summary statistics.

Morphometric features were grouped into four categories: size (nuclear area, equivalent diameter, circumference, and nuclear area coefficient of variation), shape (formfactor and Feret diameters), texture (roughness), and density (density, intensity, and brightness).

For nuclear size measurements, nuclear area and circumference were directly measured, providing a simplified size approximation that disregards shape. The nuclear area coefficient of variation (NACV) was calculated as $\left(\frac{SD\;of\;nuclear\;area}{mean\;nuclear\;area}\right)\times 100$.

Shape parameters included the formfactor, the minimum and maximum Feret diameters, and the maximum Feret diameter measured at 90° rotation (maxFeret90). The formfactor quantifies how closely an object approximates a perfect circle, with values approaching 1 indicating high circularity. This was calculated as Formfactor = $\frac{\left(4\pi \times area\right)}{{(circumference}^{2})}$. MinFeret and MaxFeret describe the shortest and longest distances between two parallel tangents applied to the nuclear contour, reflecting the minimum and maximum nuclear expansion. A high MaxFeret and a low MinFeret suggest elongated or deformed nuclei [43]. MaxFeret90 refers to the maximal Feret diameter measured perpendicular to the original MaxFeret. The difference between MaxFeret and MaxFeret90 provides an estimate of nuclear contour symmetry, with larger differences indicating greater irregularity [44].

The density parameters included density and intensity (brightness). Sum intensity represents the total of all pixel intensity values within a selected region. Sum density (or integrated density) was defined as the cumulative density of all measured pixel values normalized by area, accounting for spatial distribution: $SumDensity=\sum \left(pixel\;intensity\times pixel\;area\right)$. This allows for comparisons between nuclei of different sizes and enables conclusions about chromatin content. Denser chromatin packing or increased DNA content results in higher sum or mean density values if imaging conditions are standardized. To account for area differences, sum density was preferred over sum intensity in this analysis, and images were captured with standardized microscope and camera settings.

Normality testing rejected the null hypothesis of a normal distribution for all parameters. Compared with non-recurring tumors, Levene’s test showed strong evidence for heterogeneity of variances across the NRTs for all measurements (*p* < 0.001). Given the non-normal distribution and heteroscedasticity, the Kruskal–Wallis test was employed as a non-parametric option to test whether the medians across the NRTs differed significantly.

## 3. Results

### 3.1. Clinical Data and Radiology

**Figure 2:**
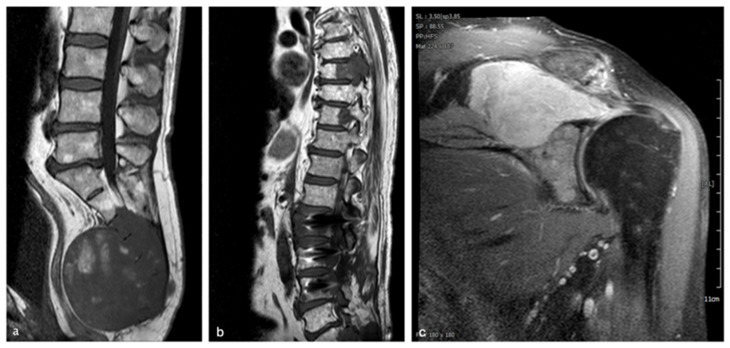
An MRI of a 70-year-old patient with sacrococcygeal chordoma. The tumor shows low signal intensity on T1-weighted images (**a**), with small foci of hyperintensity indicative of intratumoral hemorrhage. (**b**) An MRI of the same patient 7 years later, after he presented with an acute transverse lesion and received immediate radiation therapy. The sagittal T1 imaging plane shows two intraspinal lesions originating from TH6-TH8 with absolute spinal canal stenosis and possible infiltration of the myelon, as well as an increase in the size of a previously known space-occupying lesion in the lumbar spine region. The T1/TSE coronal fat-suppressed imaging plane (**c**) with intravenous contrast shows a metastasis in the processus coracoideus of the left shoulder with destruction of the glenoid; this was first diagnosed three years earlier but was left untreated because the patient had a poor cardiovascular condition.

The clinical data of all patients are summarized in Figure 1. Patients with non-recurrent tumors showed a heterogeneous age distribution, with a mean of 56.25 ± 19.11 years. In the recurrence/metastasis group, patient 10 was an outlier, diagnosed at age 70, exceeding the median age of 47.33 ± 5.9 years in this group, and raising the average age to 53.00 ± 12.30 years. His case is further distinguished by the development of multiple metastases over a seven-year period involving the lumbar and thoracic spine, as well as the coracoid process (Figure 2). The patient received systemic therapy with imatinib (2014–2016) and radiation therapy for distant metastases (shoulder/coracoid process and thoracic vertebrae) throughout 2018. Across the available images, no consistent differences were observed between patients with recurrent/metastatic chordomas and those with non-recurrent chordomas.

### 3.2. Histology

**Figure 3:**
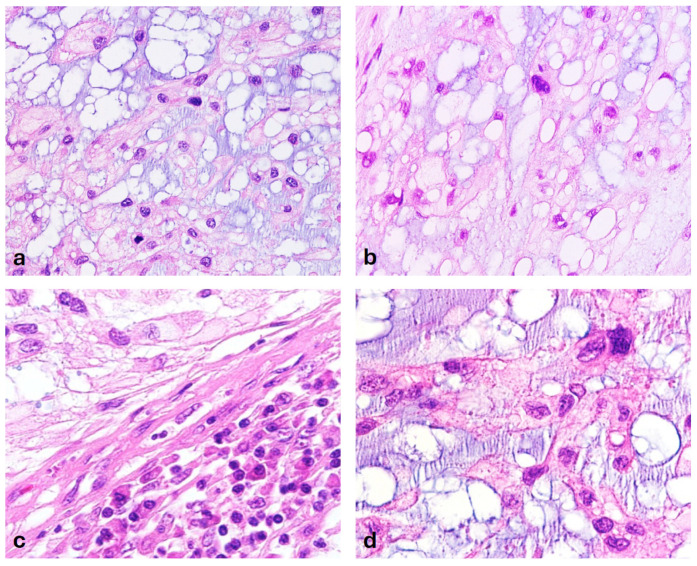
Primary tumor case 8. (**a**) Typical appearance of conventional chordoma with physaliphorous cells (H&E 400×). Histologically, no differences can be observed between the primary tumor (**a**) and the last long-term recurrence after 16 years (**b**), (H&E 400×). (**c**) Enlarged tumor cell nuclei with an irregular shape and prominent nucleoli (upper left corner) stand out in comparison to lymphocytes in the lower right corner with round, smaller nuclei, as well as adjacent fibroblasts with regular, spindle-shaped nuclei (H&E 600×). (**d**) Clusters of tumor cell nuclei with prominent coarse chromatin and markedly irregular nuclear membrane with folds and indentations, as well as heterochromatin detached from the nuclear envelope (H&E 800×).

All tumors investigated in this study—both primary and recurrent—were conventional chordomas, with intralesional fibrous septa containing fibroblastic cells and collagen fibers, which are typical defining morphologic features [45]. Such septa were present to varying degrees in all samples. Overall, their share of tissue was relatively low. The estimated tumor purity exceeded 80% in all specimens. In most cases, the characteristic myxoid pattern predominated. This pattern is marked by abundant extracellular spaces that give the superficial impression of intracellular vacuoles—a phenomenon that originally led to the concept of “physaliphorous” cells [4]. The solid subtype of myxoid chordoma, as described by Naka et al. in 2003, was not observed in our cohort [28]. A histological survey did not reveal any differences in the histological appearance between the primary tumor and fourth recurrence in case 8 after 16 years (Figure 3a,b). The degree of nuclear pleomorphism appeared to be broadly comparable between the primary tumors and recurrent samples (Figure 3a,b). The distant recurrence (metastasis) also exhibited a comparable histological pattern. At high-power magnification, some chordoma cell nuclei exhibited significant pleomorphism, which is a distinctive cytological characteristic of this tumor entity [28,29]. This pleomorphism was particularly evident when compared with the nuclei of adjacent lymphocytes and fibroblasts (Figure 3c). In some tumor areas, pleomorphic nuclei with coarse chromatin were observed, along with membrane dents and folds and detachment of heterochromatin from the nuclear envelope (Figure 3d).

### 3.3. Immunohistochemistry

**Figure 4:**
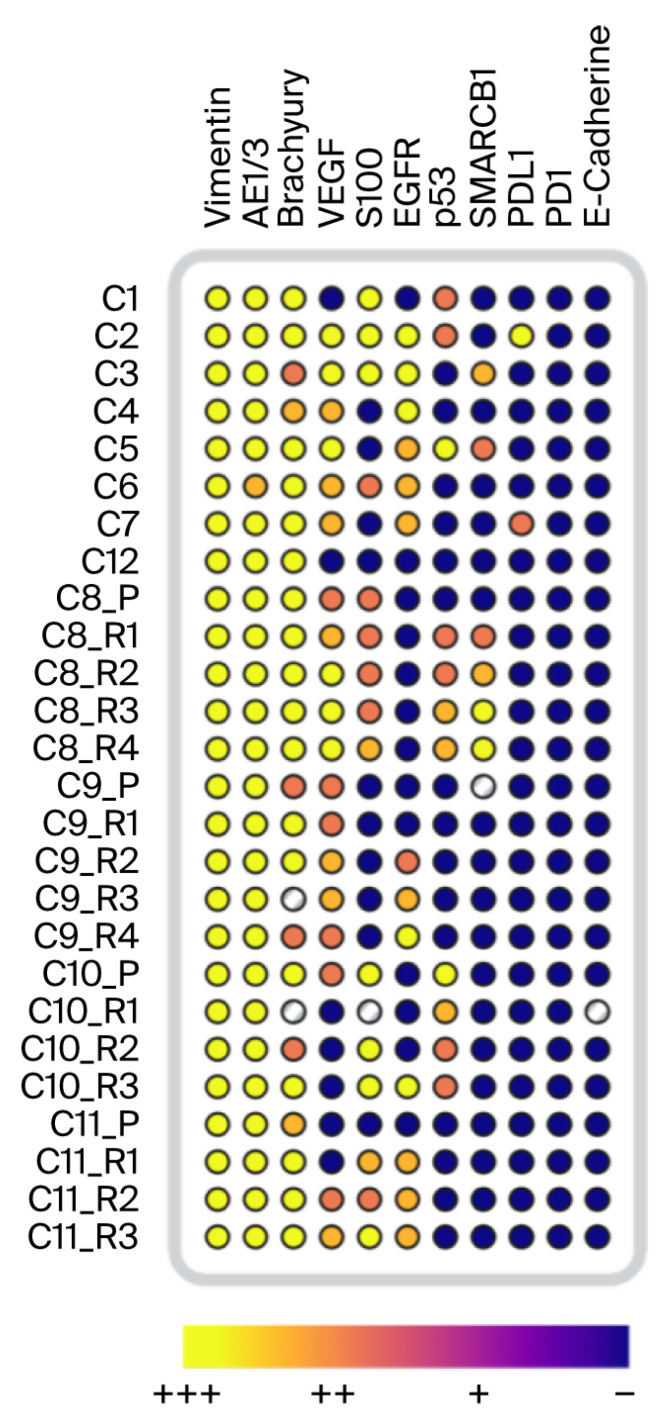
Well plate heatmap of all immunohistochemical results, categorized as strongly positive (+++), positive (++), slightly positive (+), and negative (−). Wells without color represent samples with methodical difficulties in staining.

The immunohistochemical results are summarized in Figure 4. They were evaluated using a semiquantitative scoring system [46]. All samples stained positive for vimentin and cytokeratin AE1/3, with no observable differences in expression between primary and recurrent tumors. VEGF staining among the NRT samples was heterogeneous: 20% were negative, 30% were strongly positive, and 50% were moderately positive. Notably, case 10 showed weak VEGF expression in the primary tumor but no detectable expression in subsequent recurrences. EGFR showed a similar heterogeneous pattern among the NRTs. Sixty percent of the NRTs stained positive for S100. In contrast, three out of four recurrent cases demonstrated increases in VEGF, EGFR, and S100 expression over time. SMARCB1 loss has recently been associated with poorly differentiated chordomas [47,48], and partial loss has been observed in conventional chordomas [49]. Partial nuclear protein loss was observed in some NRTs in our cohort. However, SMARCB1 immunoreactivity was reduced in recurrent cases, most notably in case 8, where SMARCB1 expression was retained in the primary tumor with subsequent progressive loss. All samples stained positive for brachyury.

**Figure 5:**
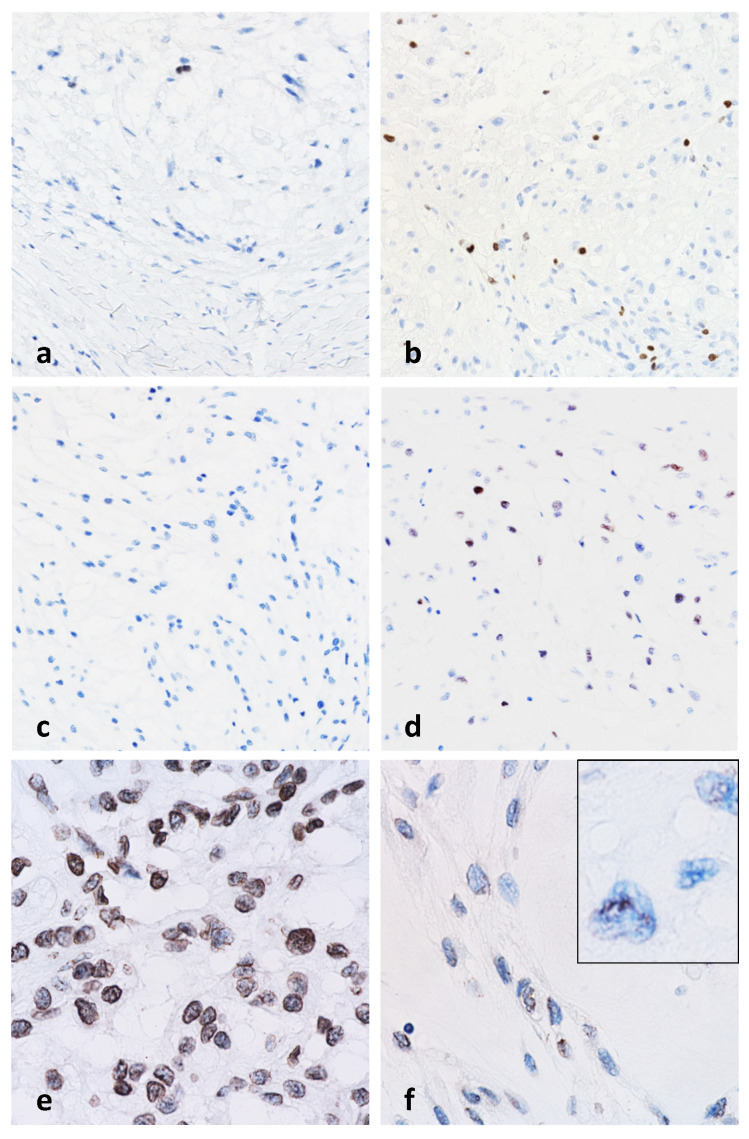
(**a**) Low Ki-67 expression index in the primary tumor of case 8 (200×). (**b**) High index in the fourth recurrence after 16 years (200×). (**c**) Occasional p53-positive nuclei in the primary tumor (200×). (**d**) Higher p53 expression in the last recurrence. (**e**) Strong expression of lamin A/C at the nuclear envelope of nuclei from the primary tumor (400×). (**f**) Decreased lamin A/C staining in pleomorphic nuclei after 16 years. Spatial disintegration of lamin A/C can be observed (inset 600×).

The Ki-67 proliferation index increased from the primary tumors to the last recurrences by a mean of 5.9 ± 4.4% (Figure 5a,b). The proliferation indices in the NRT group (4.6 ± 3.5%) were lower than those in the recurrent samples (7.1 ± 3.6%). The Ki-67 index in the primary tumors of recurrent cases (4.1 ± 3.7%) was comparable to that in the NRTs, but proliferation increased markedly in the recurrences. p53 immunoreactivity was variable, and positive nuclei were observed in approximately half of the NRT samples and recurrent cases. Case 8 exhibited an increased staining intensity over time, whereas case 10 lost p53 expression. As depicted in Figure 5c,d, p53 positivity was generally lower in primary tumors (<1%) than in recurrent tumors (>10%).

Lamin A/C staining showed strong expression at the nuclear envelope, particularly in the primary tumor of case 8 (Figure 5e). In the fourth recurrence after 16 years, ≤ 50% of the nuclei remained positive, and staining intensity was markedly reduced. (Figure 5f). Using the four-tier scoring system described by Kusano et al. [46], the primary tumor was scored as (+++) (strong expression in the majority of nuclei) and the fourth recurrence as (+) (weak expression). Furthermore, the pleomorphic nuclei in the last recurrence showed spatial disintegration of the lamin A/C component, with only lamin remnants remaining in the nuclear envelope (Figure 5f inset 600×).

### 3.4. Morphometry

**Figure 6:**
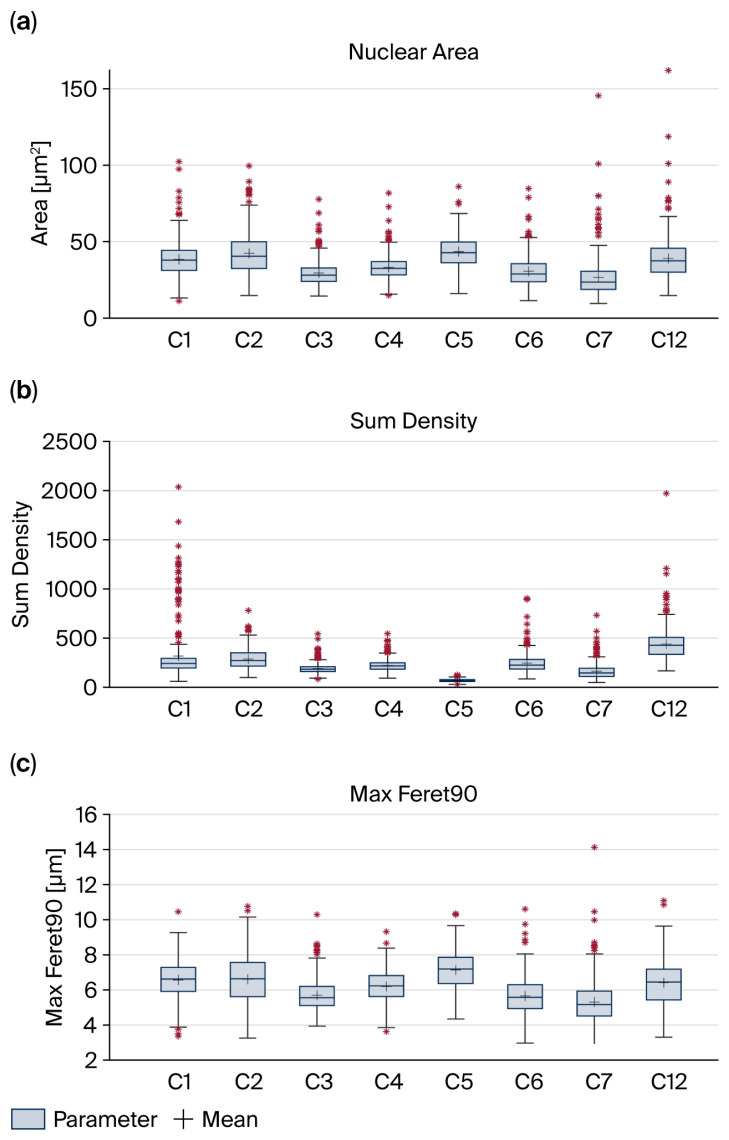
Boxplots of nuclear area as an example of a size parameter (**a**), of density (**b**) and MaxFeret90 as an example of a shape parameter (**c**). All parameters show a heterogenous distribution of measurements in non-recurrent tumors.

The NRTs displayed a heterogeneous distribution, with most samples showing pronounced intertumoral variability in morphometric parameters (Figure 6). Across specimen-level medians, variability was evident in both size- and density-related features and was summarized descriptively using range, IQR, and the between-tumor coefficient of variation. Between NRTs, most nuclear size, density, and shape parameters showed high-to-moderate intertumor variability. Nuclear formfactor was highly conserved (CV 2.28%). A slight trend toward increased asymmetry was observed.

**Figure 7:**
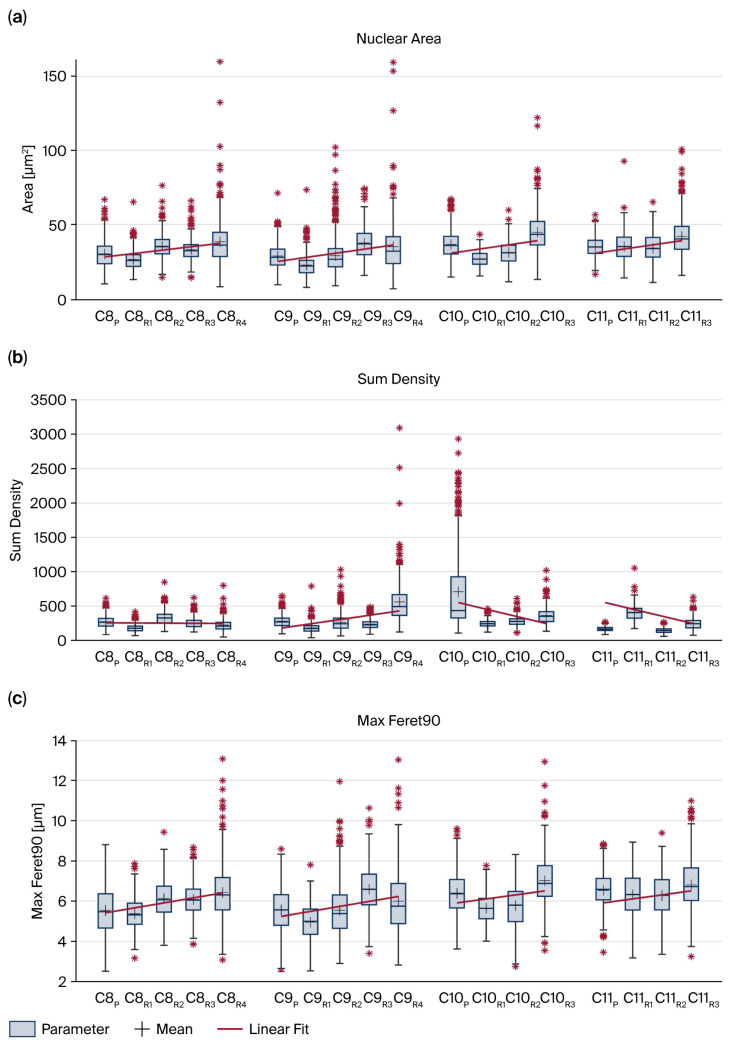
Boxplots with linear fit of nuclear area as a size parameter (**a**), density (**b**), and Max Feret90 as a shape parameter (**c**) in recurrent cases. Most patients show a highly significant increase in size over time, as well as in all feret measurements, indicating increasing deviances in shape. No trend in the density (**b**) of samples can be observed over time. The distant recurrences in case 10 revealed a pattern similar to the local recurrences.

Longitudinal morphometric trajectories differed between recurrent cases, with pronounced changes in some patients (e.g., case 8) and minimal changes in others (e.g., case 11). Case 8 showed highly significant changes (*p* < 0.001) in size-related parameters (nuclear area, equivalent diameter, and circumference) and density measures, except for the area and diameter between the second and third recurrence (Figure 7a). All density measurements differed significantly (Figure 7b). Furthermore, MaxFeret and formfactor also changed significantly, whereas MinFeret and MaxFeret90 showed significant changes only between the first and second recurrences (Figure 7c). Case 9 demonstrated similar results, with significant differences in all size parameters, except for the circumference between the third and fourth recurrence (*p* = 0.299) (Figure 7a). The shape parameters again showed analogous results, except for MaxFeret between the third and fourth recurrences (Figure 7c). Case 10, which clinically stood out for hematogenous metastasis, showed significant changes in all parameters, including size and density (Figure 7a,b). Three exceptions were noted among the shape parameters: MinFeret (*p* = 0.435) and MaxFeret90 (*p* = 0.715) between the first and second recurrence and formfactor between the second and third recurrence (Figure 7c). Morphometric trajectories of distant metastatic lesions in case 10 were comparable to those of local recurrences. However, given this single metastatic case, this observation is descriptive.

Contrary to these results, in case 11, non-significant findings were obtained for all size parameters (Figure 7a), and only some significant differences were observed in the formfactor (primary tumor vs. first recurrence) as well as in MinFeret, MaxFeret, and MaxFeret90 (second vs. third recurrence) (Figure 7c). Interestingly, sum density differed significantly across all samples in this case (Figure 7b).

The texture parameters (roughness) and additional density measures showed no consistent trends or significant differences between the NRTs and recurrent cases, nor over time within recurrences.

Finally, the Mann–Whitney U test was used to compare primary tumors from patients who later recurred with NRTs, after confirming variance heterogeneity via Levene’s test. Statistically, non-recurrent tumors do not differ significantly from primary tumors of recurrent cases. Table 1 summarizes the parameter values and *p*-values of both groups. The primary tumors in the recurrent cases had slightly smaller nuclei, a greater deviation from the circular shape, and a higher nuclear density than the non-recurrent tumors. As noted above, these differences were not apparent on the qualitative histologic assessment of the H&E slides.

Overall, nuclear morphometry was heterogeneous across non-recurrent tumors and evolved markedly over time in recurrent cases. Yet, primary tumors from patients who later recurred did not differ significantly from those of non-recurrent patients at baseline.

### 3.5. Correlation Between Morphometry and Molecular Data

To evaluate the relationship between proliferation and nuclear pleomorphism, the results of the Ki-67 index and p53 immunoreactivity were compared with those of the morphometric parameters (NACV, nuclear area, and TMB) (see Table 2).

**Table 2:** The Ki-67 index in the NRTs (mean 4.3%) was generally lower than in the RCs (overall mean of 6.5%). The primary tumors in recurrent cases showed Ki67 levels comparable to those of the NRTs (mean 4.1%), whereas the last available long-term recurrences showed higher proliferation (mean 7.1%). In all four recurrent cases, the Ki-67 index increased by a mean of 5.9% from the primary tumor to the last recurrence. p53 positivity showed a similar trend to Ki-67 in case 8, with a significant increase in recurrence rate compared with the primary tumor. The remaining recurrent cases showed low or absent p53 immunoreactivity. Only two samples exceeded the predefined 53-high threshold (>10% positive tumor nuclei). Intriguingly, in the NRTs, the change in mean nuclear area over time correlated with the percentage of p53-positive nuclei and reflected changes in the pleomorphic pattern. The TMB did not show uniform longitudinal changes across the recurrent cases. However, the overall mean TMB in the NRT cases (1.55) was lower than that in the RCs (4.75) (Mann–Whitney U, *p* = 0.025). n.s. = not sequenced. P = primary tumor. Rn = Recurrence number.

NRT/Recurrent Case	Case No.	Ki-67 Index [%]	p53 [%]	NACV	TMB
NRTs	1	4.18	2.51	31.83	0.41
	2	10.68	10.01	35.47	0.68
	3	7.53	7.43	30.32	0.09
	4	0.44	2.57	24.51	0.38
	5	2.82	24.13	24.71	0.18
	6	2.09	0.00	35.10	0.21
	7	1.67	0.17	53.43	0.12
	12	7.12	0.00	37.09	10.35
Recurrent Case 8	C8_P (primary)	1.68	0.00	30.79	0.76
	C8_R1 (first recurrence)	2.78	14.14	26.11	0.18
	C8_R2 (second recurrence)	6.38	11.21	23.33	1.71
	C8_R3 (third recurrence)	7.28	11.32	22.59	2.91
	C8_R4 (fourth recurrence)	8.98	24.98	41.54	3.44
Recurrent Case 9	C9_P (primary)	7.62	0.00	31.05	1.88
	C9_R1 (first recurrence)	3.26	0.31	33.97	8.44
	C9_R2 (second recurrence)	9.01	0.00	40.67	n.s.
	C9_R3 (third recurrence)	8.85	0.00	27.74	12.62
	C9_R4 (fourth recurrence)	7.05	0.00	49.89	0.97
Recurrent Case 10	C10_P (primary)	9.96	7.10	25.99	0.06
	C10_R1 (first recurrence)	0.08	0.01	25.78	n.s.
	C10_R2 (second recurrence)	14.69	3.45	31.44	13.88
Recurrent Case 11	C11_P (primary)	0.18	0.00	17.90	n.s.
	C11_R1 (first recurrence)	4.88	0.00	26.59	7.53
	C11_R2 (second recurrence)	9.25	0.00	27.46	8.56
	C11_R3 (third recurrence)	9.30	0.00	32.10	3.65

The samples were stratified into Ki-67-low (<5%) and -high (>5%) groups, and the mean nuclear area was compared between these groups using the Mann–Whitney U test. As shown in Table 3, the mean nuclear area in the Ki-67-high group (35.96 μm^2^) was larger than that in the Ki-67-low group (31.76 μm^2^). However, the Mann–Whitney U test yielded a *p*-value of 0.085, suggesting limited power given the sample size (Table 3). Similarly, the samples were stratified into p53-high (>10% positive nuclei) and p53-low (<10%) groups. As shown in Figure 4, half of the NRTs and all recurrences in case 8 showed strong positive staining. The p53-high samples had a higher mean nuclear area (36.42 μm^2^) than the p53-negative group (33.42 μm^2^), although the difference was not statistically significant (*p* = 0.15). The nuclear area did not differ significantly between the high- and low-TMB samples. Furthermore, the samples with high proliferation indices or high p53 positivity did not show a significantly higher tumor mutational burden.

**Figure 8:**
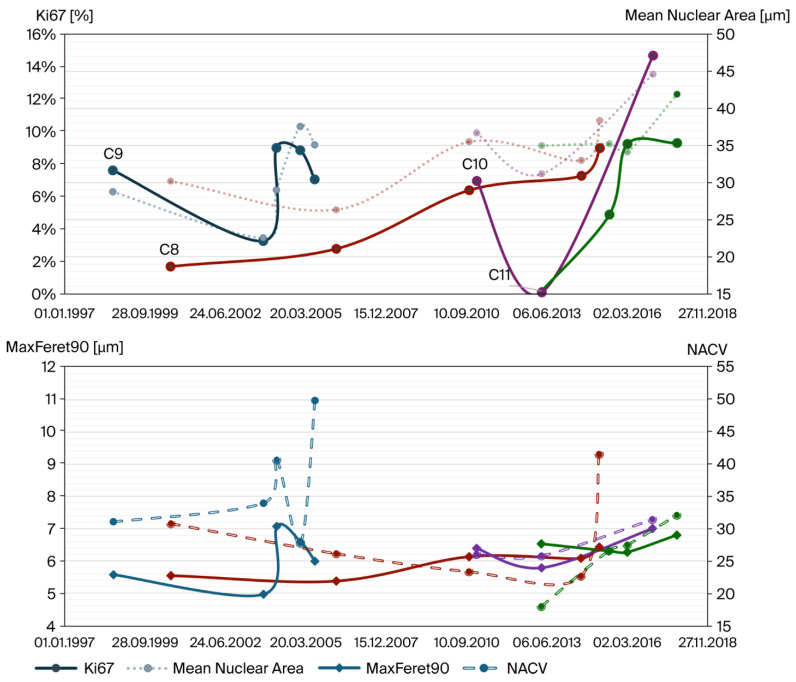
Correlation of the development of the Ki-67 index with the mean nuclear area, as well as MaxFeret90 with the NACV, over the exact timeline of recurrence development in all four recurring cases. The nuclear pleomorphic parameters correlate well with the relative proliferative activity over time in all recurrent cases.

When plotted against the elapsed time between the primary tumor and subsequent recurrences, the Ki-67 indices and mean nuclear area showed similar trajectories (Figure 8), consistent with the previous comparisons.

The coefficient of variation of nuclear area (NACV) reflects nuclear size heterogeneity and correlates well with asymmetry-related measures, as reflected in MaxFeret90. These two parameters showed similar progression, with a tendency to increase over time. A high NACV reflects greater variability in nuclear size, while an increased MaxFeret90 indicates an elongated and irregular nuclear shape. Taken together, these parameters can serve as quantitative markers of nuclear pleomorphism.

**Figure 9:**
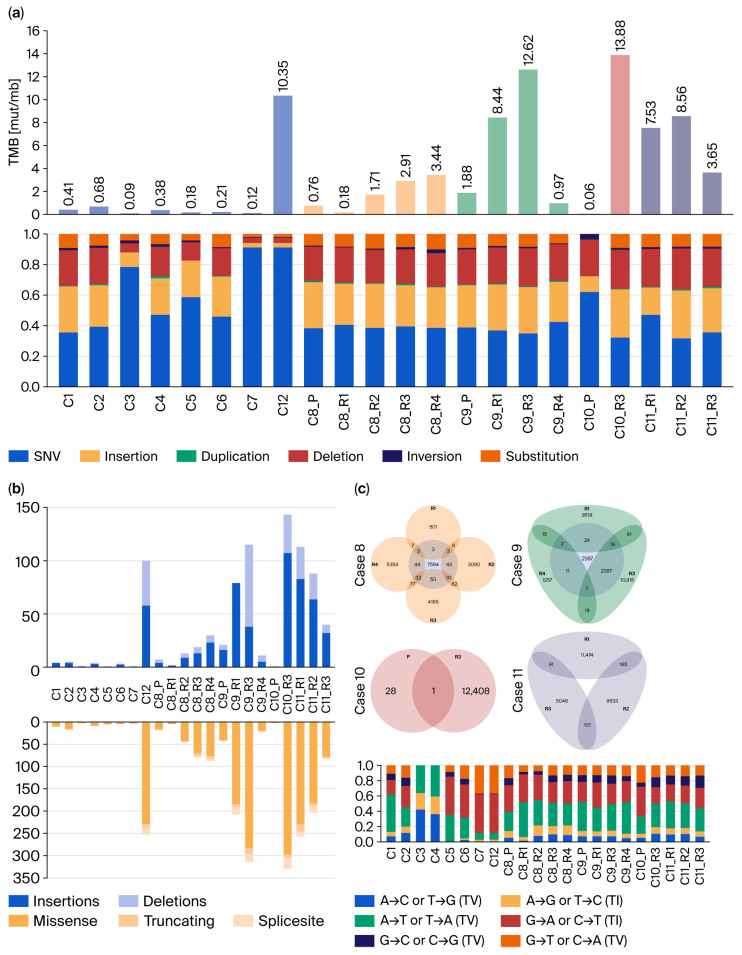
(**a**) Correlation of the tumor mutational burden (TMB) with the mutational distribution of all samples. Except for case 12, all NRTs displayed a lower TMB than the long-term recurrences or metastases in the RCs. The mean TMB in the recurrent cases (4.75 Mut/Mb) was significantly (*p* = 0.02 via Mann–Whitney U) higher than that in the NRTs (1.55 Mut/Mb). Case 8 showed a continuous increase in TMB across its recurrences, whereas no trend was observed in the other RCs. Mutational events were predominantly represented by single-nucleotide variants (SNVs) and insertions, as well as deletions (InDels). (**b**) Insertions occurred more frequently than deletions. SNVs were dominated by missense mutations. The transition (TI) and transversion (TV) graph summarizes the spectrum of SNVs detected in this dataset. Substitutions within the same nucleotide class are classified as transitions (TIs), and substitutions between nucleotide classes are classified as transversions (TVs). (**c**) Venn diagram of all mutations found in the recurrent cases before filtering, highlighting their shared mutations (classified as alterations with the same amino acid change at the same position).

An analysis of the TMB and mutational spectrum (Figure 9a) did not reveal clear differences between the NRTs, primary tumors in recurrent cases, and long-term recurrences or metastases. In an exploratory comparison, the mean TMB in the long-term recurrences (4.75 Mut/Mb) was higher than that in the NRTs (1.55 Mut/Mb; *p* = 0.02 via Mann–Whitney U). However, given the small number of sequenced samples and heterogeneous case-specific trajectories, this finding should be interpreted cautiously. A detailed examination of the mutational spectrum (Figure 9b) revealed that the ratio of InDels to SNVs was consistent in all samples. This reproducibility across independent sequencing runs and experimental batches supports the internal consistency of the variant-calling workflow and provides an additional, qualitative measure of internal quality control. A closer examination of the SNVs showed a missense-dominated mutational composition. A transition–transversion plot was used to further characterize the SNV spectrum observed across this dataset. The Venn diagram in Figure 9c shows a modest number of shared mutations throughout serial long-term recurrences in the same patient.

Overall, we did not observe a consistent association between TMB and nuclear morphometric parameters across cases. Morphometric remodeling and proliferative changes occurred despite heterogeneous and patient-specific TMB trajectories.

## 4. Discussion

Chordomas are rare malignant tumors of the axial skeleton with high recurrence rates despite their low-grade histological appearance [50]. Reliable prognostic markers to identify patients at risk of recurrence remain scarce, complicating therapeutic stratification and follow-up [51,52,53,54].

In the last several years, morphometric analysis of nuclear features has emerged as a promising tool for assessing tumor behavior in various cancer types [19,20]. By quantifying subtle changes in nuclear size, shape, and density, this technique may capture histological correlates of biological aggressiveness that are not readily apparent on routine microscopy [55]. Morphometric abnormalities are pivotal criteria for the histopathologic diagnosis of malignancy [11,30], with size, shape, chromatin structure, and particularly irregularities of the outer nuclear membrane automatically taken into consideration [31,32]. Several studies have demonstrated that nuclear morphometric alterations may distinguish aggressive tumor behavior, including metastatic potential or recurrence, in other malignancies [24,25,26,27,44]. Despite these promising findings, such approaches have not yet been systematically applied to chordoma [45].

To investigate whether nuclear pleomorphic changes are associated with tumor progression in chordoma, we quantified nuclear features using nuclear morphometry. We integrated these data with immunohistochemical findings and tumor mutational burden derived from whole-exome sequencing.

Our analysis of non-recurrent tumors (NRTs) revealed considerable intertumoral variability in nuclear morphology. Chordomas are known for their pronounced intratumoral heterogeneity, which may even be reflected in morphologically stable cases in different clonal regions with variable morphometric profiles [56,57]. To minimize sampling bias, multiple regions per tumor were chosen at random and analyzed. Nonetheless, the observed variability may reflect biological differences in tumor cell populations, anatomical site-specific factors, or early-stage subclonal diversification that does not necessarily result in recurrence. These results highlight the need to contextualize morphometric features within the overall tumor architecture and to interpret single-parameter readouts cautiously when assessing morphometry for risk.

In tumors obtained from patients with multiple long-term recurrences, we observed consistent changes over time. Most notably, size-related parameters, including nuclear area, equivalent diameter, and circumference, tended to increase across successive recurrences. These changes were accompanied by increasing nuclear asymmetry, reflected in altered formfactor and feret-based measurements. These morphometry transformations may reflect changes in clinical presentation, phenotypic shifts, and progressive disease evolution, as well as increased genomic instability. Furthermore, increasing NACV across recurrences supports nuclear pleomorphism as a quantitative morphological correlate of tumor heterogeneity and potentially aggressive behavior [10].

As key components of the nuclear envelope, lamins are crucial for nuclear mechanics and shape [58,59,60]. They correlate with cytologic malignancy criteria and are involved in cancer progression [61]. Altered lamin A/C expression has been associated with adverse tumor behaviors in several malignancies, including metastasis and poor prognosis [62,63,64]. Correspondingly, we observed a negative correlation between nuclear envelope lamin A/C expression and the shape and size parameters. Similar findings were reported in Ewing sarcoma [65]. Furthermore, the pleomorphic nuclei in the last recurrence revealed abnormal cytoplasmic localization of the lamin A/C component, with only lamin remnants remaining in the nuclear envelope. This spatial disintegration correlated with the increase in nuclear pleomorphism over time, supporting a role of lamin A/C in nuclear mechanical stability. In line with the literature, the abnormal localization of lamins may contribute to pleomorphic nuclear remodeling and link morphometric alterations to nuclear envelope integrity. [66]. Rim disruption was assessed qualitatively on representative sections and should be interpreted as descriptive evidence pending quantitative validation in larger cohorts.

Overall, these findings suggest that recurrence evolution is accompanied by measurable nuclear remodeling, offering a morphological window into the biological shifts that drive disease progression. Among the recurrent cases, patient 10 stood out due to the development of multiple hematogenous metastases over seven years. Here, the term metastases denotes distant recurrences, which are defined as metastases following an initial disease-free interval [67]. This clinical course is compatible with a particularly aggressive disease phenotype, potentially reflecting a unique tumor biology or patient-specific risk factors [68]. In this patient, we observed the largest rise in the TMB across serial samples and the highest Ki-67 index in a metastatic lesion (14.7%), together with pronounced morphometric changes, supporting the hypothesis that advanced disease may be associated with accelerated proliferation, measurable nuclear remodeling, and molecular changes. Furthermore, this may indicate a higher degree of malignancy in long-term hematogenous metastatic chordoma tissue than in local recurrences. A similar phenomenon has been reported in long-term metastases of breast cancer [35].

However, given the small cohort size, these observations remain hypothesis-generating and require confirmation in larger cohorts of metastatic chordoma. Despite the marked clinical divergence, radiological assessment did not reveal consistent distinguishing features, highlighting the limitations of conventional imaging techniques in predicting tumor behavior [50,69] and reinforcing the potential value of quantitative nuclear profiling in identifying aggressive trajectories.

Primary tumors from patients who later developed multiple long-term recurrences did not differ from non-recurrent tumors in several morphometric parameters. They exhibited smaller nuclei with greater asymmetry and higher density. The increased nuclear density observed in primary tumors may reflect denser chromatin packaging, increased DNA content, or altered transcriptional activity—features often associated with biological aggressiveness in other malignancies [70]. One possible explanation for the observed smaller nuclei is that these tumors may harbor subclonal populations with highly compacted chromatin [61], resulting in a reduced nuclear size despite increased functional activity [71]. Alternatively, smaller nuclei with irregular contours could reflect a distinct differentiation state predisposing these tumors to recurrence [30]. Similar morphometric patterns have been reported in prostate and thyroid carcinomas, where smaller, denser nuclei with higher asymmetry were associated with adverse outcomes despite their otherwise low histological grade [17,30,44]. Overall, these findings provide preliminary evidence of baseline morphometric differences between primary tumors from patients with non-recurrent disease and those from patients who later developed recurrences or metastases. However, given the small cohort size and the descriptive design, these observations are hypothesis-generating and insufficient for clinical risk stratification. Validation in larger, independent cohorts with patient-level modeling is required.

Together, morphometry and immunohistochemistry suggest a progressive transformation in recurrent chordoma with increasing nuclear size/asymmetry paralleling higher Ki-67 indices, consistent with a more proliferative phenotype [72,73,74]. Case 8 showed increased p53 and partial SMARCB1 loss, a pattern linked to more aggressive chordoma subtypes, although partial loss has also been reported in conventional chordoma [48,49]. However, Maioli et al. found mosaic or clonal partial loss of SMARCB1 in conventional spinal chordoma, which could explain the weak staining or loss of expression in NRTs [49]. Across RCs, recurrences showed heterogeneous changes, including increases in EGFR, VEGF, and S100 reactivity, or altered p53 expression. VEGFA expression has previously been associated with shorter progression-free survival [75]. E-cadherin expression was negative in all samples, indicating a chordoma-typical mesenchymal switch profile with the upregulation of N-cadherin and downregulation of E-cadherin, which could be caused by the repression of CDH1 through promoter methylation. Consistent with prior observations in conventional chordoma, PD-L1 and PD-1 were negative in most patients (0%/17%), suggesting a limited role of these markers in the immune microenvironment of chordoma [76]. However, the diversity of staining patterns across cases suggests that chordoma recurrence is not driven by a uniform set of molecular events but rather by heterogeneous biological trajectories [31,77]. Taken together, the pleomorphic changes and immunohistochemical findings support the interpretation that recurrent-stage tumors exhibit higher proliferative activity and potentially increased genetic instability [30,78,79]. These findings reinforce the value of nuclear morphometry as a reproducible and quantifiable tool that can capture biologically relevant changes, even in the absence of consistent immunohistochemical alterations.

Morphonuclear profiling in long-term recurrences was integrated with the tumor mutational burden (TMB) as a measure of molecular genetic complexity. The TMB is defined as the total number of somatic nonsynonymous mutations present in the exome of the cancer genome [78]. In clinical oncology, it is used to predict the response to immune checkpoint therapy [80,81]. It can also be used as a measure of the molecular genetic complexity of malignant tumors. The literature indicates that nuclear morphology is associated with genomic instability metrics across multiple cancer types [21]. The relationship between TMB and nuclear morphology is supported by several studies demonstrating the potential to predict TMB from histopathological images, for example, in lung adenocarcinoma [82] and colorectal cancer [83,84]. Samples from non-recurrent cases showed a significantly lower TMB than samples from recurrent cases (*p* = 0.02). In a previous study, the TMB in chordoma was reported to be generally low, with a range of 0.05–7.68 mut/mb [9,85,86], but only one recurrence paired with its primary tumor was investigated. The time to first recurrence (TTFR) ranged from 3 to 36 months, with a median of 8 months, excluding long-term recurrences. Investigations into the TMB of other solid tumors yielded different results: The average tumor mutational burden observed in triple-negative breast cancer was 7.6 mut/mb, with 8.3 mut/mb in recurrent tumors and 7.2 mut/mb in primary tumors that did not recur [87]. Contrarily, Wei et al. found a positive correlation between the time of relapse and increased TMB in glioblastomas [56]. Metastatic tumors are pathogenetically related but differ from recurrent tumors and should be assessed differently. In non-small-cell lung cancer, the molecular genetic pattern of brain metastases was found to differ from that of primary tumors [88].

Among the recurrent cases, patient 10 stood out due to the development of multiple hematogenous metastases over seven years. Here, the term metastases denotes distant recurrences, which are defined as metastases following an initial disease-free interval [67]. This clinical course is compatible with a particularly aggressive disease phenotype, potentially reflecting a unique tumor biology or patient-specific risk factors [68]. In this patient, we observed the largest rise in the TMB across serial samples and the highest Ki-67 index in a metastatic lesion (14.7%), together with pronounced morphometric changes, supporting the hypothesis that advanced disease may be associated with accelerated proliferation, measurable nuclear remodeling, and molecular changes. Furthermore, this may indicate a higher degree of malignancy in long-term hematogenous metastatic chordoma tissue than in local recurrences. A similar phenomenon has been reported in long-term metastases of breast cancer [35].

However, given the small cohort size, these observations remain hypothesis-generating and require confirmation in larger cohorts of metastatic chordoma.

In our study, the TMB in recurrent cases showed heterogeneous, patient-specific behavior and did not exhibit a uniform temporal increase throughout the development of long-term recurrences and metastases over a time span of 7 to 16 years. Several investigations have shown that the TMB can differ between primary tumors and recurrences [89]. A comprehensive genome-wide comparison of primary and metastatic solid tumors reported that the majority of cancer types exhibited either moderate genomic variations (e.g., lung adenocarcinoma) or relatively conserved genomic patterns (e.g., ovarian serous carcinoma) [90]. The same study also described global genomic differences between primary and metastatic cancers: metastatic tumors often showed higher clonality and lower intratumor heterogeneity than primary tumors. Overall, these findings suggest that TMB alone is not a consistent marker of progression in this cohort, and that tumor progression is more closely linked to clinically relevant driver genes. Furthermore, in our study, correlation analyses between morphometric parameters and TMB were underpowered due to the small number of independent cases and missing WES data at certain timepoints. Thus, associations should be interpreted as exploratory.

In our cohort of long-term recurrent chordomas, nuclear morphometric parameters, including size and shape factors, showed statistically significant changes over time. Although these alterations were moderate in absolute terms, they reflected the morphometric nuclear changes in recurrences over time. They correlated well with decreasing lamin A/C expression, which may contribute to reduced nuclear mechanical stability. The inverse correlation between lamin A/C expression at the nuclear envelope and nuclear area/shape parameters offers a plausible mechanistic link between the observed increase in nuclear irregularity and the higher proportion of pleomorphic nuclei in recurrences [91]. This is compatible with the overall modest molecular evolution captured by the TMB. To the best of our knowledge, no other studies have integrated longitudinal quantitative morphometry with genomic profiling in long-term recurrent/metastatic chordoma. Our comparative analysis provides exploratory evidence linking morphonuclear changes with molecular features in long-term recurrences and metastases. These findings may contribute to a better understanding of recurrence formation and metastasis in chordoma and guide future studies on biomarker-guided stratification and therapeutic development.

This study has three main limitations that should be considered when interpreting the results.

First, Tissue samples were obtained from archival material spanning different time periods, making them susceptible to variations in processing methods and preanalytical conditions. Differences in fixation, section thickness, or surface quality, staining protocols, and storage conditions may affect color distribution and thereby introduce variability in pixel-based parameters such as density, intensity, and roughness, potentially leading to information bias. However, fully automated segmentation and batch-standardized staining protocols could enhance reproducibility in future analyses.

Second, the sample size in our study was limited—as it is in many chordoma studies due to the tumor’s rarity—limiting the generalizability of our findings. This also limits statistical power to evaluate rare features, such as specific immunohistochemical expression patterns or clinical characteristics, such as metastasis development. Statistical comparisons in the recurrent group are underpowered and susceptible to sampling variability. Therefore, longitudinal findings must be interpreted descriptively and carefully. Confirmation in larger, independent cohorts would increase statistical power.

Third, the recurrence group showed some clinical heterogeneity: Three patients had serial local recurrences, whereas patient 10 developed distant metastatic lesions. Accordingly, observations related to metastatic lesions are interpreted descriptively and should be validated in larger metastatic chordoma cohorts.

Semiautomated methods—typically involving manual tracing of nuclei on digitized histologic images—have long been standard approaches for nuclear morphometry assessment [39]. Our study used a single trained investigator who was blinded to sampling status and patient data and conducted all measurements. This approach reduced inter-observer variability by avoiding differences between multiple raters; however, external reproducibility should be evaluated in future multi-rater or fully automated pipelines.

While fully automated image analysis pipelines are not yet standard in routine histopathology, advances in artificial intelligence (AI) and digital pathology are expected to accelerate their adoption in the near future [92,93]. As manual tracing of nuclei for morphometry analysis would be too time-consuming in a diagnostic clinical setting and may introduce observer-dependent variability, rapid advances in AI could facilitate quantitative and qualitative nuclear assessments and contribute to more reliable cancer diagnostics [94]. This study could provide data on nuclear development in long-term recurrences, and tracing results could be used for training in future AI-based studies, given the high number of measurements per sample.

## 5. Conclusions

In this study, quantitative nuclear morphometry captured measurable phenotypic remodeling during long-term recurrence evolution and metastasis. Non-recurrent chordomas showed substantial intertumoral variability, whereas serial samples from recurrent/metastatic cases demonstrated consistent longitudinal changes, most predominantly in size- and shape-related parameters, accompanied by higher proliferative activity in recurrent lesions. However, primary tumors from patients who later recurred did not differ from non-recurrent tumors and showed comparable qualitative histology.

Integration of morphology with immunohistochemistry and whole-exome sequencing indicated that morphonuclear remodeling occurs alongside heterogeneous molecular trajectories. The TMB remained low overall but varied across patients and timepoints and was higher in recurrent tumors, emphasizing that mutational load alone cannot serve as a marker of progression in chordoma. Lamin A/C expression decreased over time within recurrences and was inversely associated with nuclear irregularity, indicating a mechanistic link between the loss of nuclear envelope integrity and increased pleomorphism.

Collectively, these findings suggest that quantitative nuclear morphometry, complemented by immunophenotypic and genomic profiling, can capture recurrence-associated phenotypic remodeling in chordoma. Larger, independent cohorts are required to validate these observations, determine their clinical utility, and enable the development of robust patient-level predictive AI models that could investigate how morphonuclear remodeling relates to driver events and potential therapeutic vulnerabilities in long-term recurrent and metastatic chordoma.

## Figures and Tables

**Table 1:** Mean measurements ± standard deviation for pleomorphic parameters in non-recurrent tumors and primary tumors in recurrent cases. A *p*-value of <0.05 is considered statistically significant. Non-recurrent tumors do not differ significantly from primary tumors of recurrent cases.

Nuclear Parameter	NRTs (*n* = 8)	Primary Tumors in RCs (*n* = 4)	*p*-Value (Mann–Whitney U)
Nuclear Area [µm^2^]	34.73 ± 5.91	32.15 ± 3.92	0.570
Equivalent Diameter [µm]	6.54 ± 5.57	6.32 ± 0.43	0.570
Circumference [µm]	22.310± 2.11	21.60 ± 1.11	0.570
Formfactor	0.91 ± 0.02	0.89 ± 0.03	0.343
MinFeret [µm]	5.86 ± 0.54	5.57 ± 0.54	0.368
MaxFeret [µm]	8.45 ± 0.76	8.36 ± 0.28	0.933
MaxFeret90 [µm]	6.19 ± 0.57	5.89 ± 0.59	0.368
Density	238.23 ± 103.86	336.26 ± 219.07	0.808

**Table 3:** All samples were stratified into Ki-67-, p53-, and TMB-high and low groups. The cut-offs for the high group were as follows: Ki-67 > 5%, p53 staining > 10%, and TMB > 6.5 Mut/Mb (=75th percentile). The Ki-67-high group had a higher mean nuclear area than the Ki-67-low group and a slightly higher TMB. The samples with higher p53 positivity also had a higher mean nuclear area but a lower tumor mutational burden. However, none of the results were statistically significant, as determined using the Mann–Whitney U test. The samples with high TMB did not differ from those with low TMB in nuclear area.

Measurement		Mean Measurement	Mean Nuclear Area [µm]	*p*	TMB	*p*
Ki-67 index	low	2.19% (*n* = 10)	31.76	0.085	2.93 (*n* = 8)	0.142
	high	8.62% (*n* = 15)	35.96		4.37 (*n* = 14)	
p53 positivity	low	1.27% (*n* = 19)	33.42	0.155	4.37 (*n* = 16)	0.438
	high	15.97% (*n* = 6)	36.31		1.51 (*n* = 6)	
TMB	low	1.13 Mut/Mb (*n* = 16)	34.08	0.531	-	-
	high	10.23 Mut/Mb (*n* = 6)	35.04		-	

## Data Availability

The original contributions presented in this study are included in the article. Further inquiries can be directed to the corresponding authors. The raw data supporting the conclusions of this article will be made available by the authors on request.

## Abbreviations

The following abbreviations are used in this manuscript:

AI	Artificial intelligence
AltAF index	Alternate allele friction index
BNCT	Benign notochordal cell tumor
dbSNP	Single-Nucleotide Polymorphism Database
DNA	Deoxyribonucleic acid
EDTA	Ethylenediaminetetraacetic acid
EGFR	Epidermal growth factor receptor
FFPE	Formalin-fixed, paraffin-embedded
GRCh37	Genome Reference Consortium Human Build 37
H&E	Hematoxylin and eosin
InDel(s)	Insertion(s) and deletion(s)
Ki-67	Ki-67 proliferation index
MAF	Minor allele frequency
MATLAB	Matrix Laboratory (MathWorks software)
MaxFeret	Maximum Feret diameter
MaxFeret90	Maximum Feret diameter measured at 90° rotation
Mb(s)	Megabase(s)
MinFeret	Minimum Feret diameter
Mut/Mb	Mutations per megabase
NACV	Nuclear area coefficient of variation
NGS	Next-generation sequencing
NRT(s)	Non-recurrent tumor(s)
PD-1	Programmed cell death protein 1
PD-L1	Programmed death-ligand 1
Q-Q	Quantile–quantile (plot)
RC(s)	Recurrent case(s)
SCNA(s)	Somatic copy-number alteration(s)
SD	Standard deviation
SMARCB1	SWI/SNF-related, matrix-associated, actin-dependent regulator of chromatin, subfamily B, member 1
SNV(s)	Single-nucleotide variant(s)
TMB	Tumor mutational burden
TTFR	Time to first recurrence
VAF	Variant allele frequency
VEGF(A)	Vascular endothelial growth factor (A)
WES	Whole-exome sequencing
WHO	World Health Organization

## Nomenclature

The following clones were used in this study:

25/BAF47	SMARCB1 clone
315M-95	PD-1 clone
453M-95	p53 clone, DO7 designation
AE1/3	Pancytokeratin clone (PCK)
E30	EGFR clone
EP4520-16	Lamin A/C clone
EP700Y	E-cadherin clone
EPR18113	Brachyury clone
M3653	PD-L1 clone
MIB-1	Ki-67 clone
VG-1	VEGF clone
Vim384	Vimentin clone
Z0331	S100 antibody identifier
